# On the Treatment of Wounds Received during Dissection

**Published:** 1825-05

**Authors:** John Shaw

**Affiliations:** Lecturer in Anatomy, &c.


### Art. III.—*On the Treatment of Wounds received during Dissection.* By John Shaw, Lecturer in Anatomy, &c.

It is not my intention to enter into a discussion concerning the different views that have been lately taken of this very important question: I shall confine myself to a brief account of the mode of treatment which I have pursued in cases where certain symptoms have followed wounds received during the examination of dead bodies.

The profession is much indebted to Dr. Duncan for the collection of cases of this kind, and the valuable observations he has made on them in his paper on Diffuse Inflammation. This collection affords proofs in corroboration of a view which I have for some time entertained,—viz. that the effects of wounds received during dissection should be classed under two heads, forming cases which differ essentially from each other. The one seems to be of a specific nature, is attended with immediate danger, and is generally the consequence of examining a body a few hours after death; and, as far as my experience goes, most frequently follows dissection of the bodies of persons who have died with inflammation of some of the serous membranes. The other cases are more common, are less dangerous, and resemble in many respects those with which all who have attended to hospital practice must be familiar. I allude now to the consequences of wounds received in the dissecting-room, during the examination of parts which are putrid.

As I have twice suffered very severely from such wounds, and as several of my more immediate assistants have been similarly affected, my attention has been naturally much directed to the modes of prevention and method of cure.

The discovery of any mode by which the student may avoid the evils that have been so frequently consequent on dissection, would be very valuable; I shall therefore begin by making a few remarks upon this last affection, as I think that we have discovered means by which the operations of the dissecting-room may be carried on with comparatively little danger.

Every student, on commencing dissection, is much alarmed when he cuts or scratches his finger; but, in the rooms in Great Windmill-street, the fear of this soon ceases, and of late years serious consequences have rarely occurred. This exemption I attribute to the bodies being now invariably injected with a solution of nitre and salt, previously to their being dissected. If a part is not to be preserved as a dry preparation, there is not a single objection to the use of these salts; while the advantages, independently of the effect which it seems to have on the morbid poison, are considerable. Indeed, it is now common for students to keep a body for eight or ten weeks; and, even after this time, there is scarcely any smell, and the muscles of the limbs appear fresh and florid. During the mildest part of last winter, a young physician, after having spent some time in dissecting the neck, removed the arm from the body, wrapped it up in a dry cloth, and put it aside: he went down to Edinburgh, and, on his return about three weeks after, he found the limb in such a state of preservation as enabled him to continue the dissection for a week. It may, perhaps, be alleged that our students are not very assiduous, if they spend a month in the dissection of one limb and the side of the neck; but I have always endeavoured to encourage this mode of proceeding, for more is learnt by a thorough examination of one body than a rapid survey of many. Indeed, the high price now demanded for bodies, although in some respects inconvenient, has had certain good effects. The students acquire a better knowledge of anatomy than formerly, and they are protected from the bad consequences of dissection. The first results from the expense attending dissection inducing them to set a higher value on their opportunities; the other, from the manner the bodies are prepared for the purpose of preserving them.

Previously to our practising the plan of injecting bodies with the salt solution, very serious consequences frequently resulted from pricks or wounds, and particularly in the spring, when the students had become a little enervated from the effects of a winter in town. The only cases that have occurred during the last three or four years, have proceeded from the dissection of the ligaments, after the limb had been kept for some time in water, or in preparing bones that have been macerated; being, in short, the only occasions on which the student is subjected to putrid matter, not corrected by the solution of the nitre and baysalt.

The history of the effects of a wound made under these circumstances, is nearly the following:—The finger is scratched or pricked in the morning; there is not much pain at the time, but it gradually increases towards the evening; a little uneasiness is felt in the axilla, and next morning red lines can be perceived running up the arm. The finger is now excessively painful; there are often slight rigors, and general uneasiness; the countenance is anxious, and the tongue is sometimes furred, with head-ache; but still there is not much fever.

The finger becomes rapidly swollen and livid, so as to call for immediate attention, and the general system is so much affected that we cannot be satisfied in considering the patient as suffering merely from a local complaint.

I shall now offer a view of the general plan of treatment I have followed in such cases. The best local application is lint soaked in equal parts of laudanum and Goulard's lotion, which are to be used tepid at first. The lint should be applied in slips round the finger, and in larger pieces over the hand and up the arm; the whole of which should be kept on a pillow, the hand being on rather a higher level than the shoulder. Notwithstanding this soothing application, the pain sometimes continues so intense, that the patient begs to be allowed to try the effect of a poultice. If this be applied, a large quantity of laudanum should be put into it; but I have generally found that the patient, after a short trial of the poultice, gave it up for the wet dossils. The weight of the poultice seems to increase the irritation. In the course of the second or third day, the patient is so convinced that there is matter situated deeply, that he urges the necessity of cutting down upon it. This is a question of some difficulty. I have several times been disappointed in the expectation of finding matter, although I have cut down to the bone; but I believe it is safer to commit this error, than run the risk of allowing matter to lodge: it does occasionally form very rapidly; and if, und^r such circumstances, it be not evacuated, it keeps up a dreadful degree of irritation. It is well known that, when matter is pent up under the tendons, it quickly causes them to slough, and even the bone to exfoliate. I would therefore, were there the slightest suspicion of matter being lodged, cut fairly down to the bone. From experience in my own person, I can state how great and how immediate the relief is from its evacuation, although the operation itself is most painful. By the fifth or sixth day, if the general treatment has been judicious, the bad local symptoms will probably have subsided; but the hand should, for three weeks at least after the severer symptoms have disappeared, be carefully nursed and kept in a sling, as a sort of erysipelatous inflammation is very apt to attack it, and to run from one finger to another, and to be increased by the slightest exertion. The swellings in the axilla must not be neglected; but they have never suppurated in any case that I have seen.

The general treatment I have pursued, has been to keep the bowels in an active state, and this by a combination of calomel with the warmer purgatives, in preference to saline medicines; to keep the patient at first almost in a state of stupefaction, by laudanum and porter. This mode of treatment has been criticised; but I still recommend it, however it may be in opposition to the theories of the nature of this affection, because it appears to afford more relief than any other. While suffering severely under one of these attacks, I have, before going to bed, taken a large dose of opium, and nearly a pot of porter: in this way I have been relieved from the excessive pain, and have slept soundly, although, when I am in good health, a few drops of laudanum or a pint of porter would make me feverish and restless.

I enjoin the patient to live well, and take more wine than he has been accustomed to; and, if the arm is not hurt by motion, to keep as much as possible in the open air. When the severity of the inflammation has subsided, I advise him either to go into the country, or to take occasional short rides out of town on the outside of a coach.

In such a case as the above, (and I believe it to be the one of most frequent occurrence among medical students,) I should never advise venesection; and I doubt the propriety of applying leeches, even although the inflammation should extend up the arm: as far as I can judge from the cases that have been recorded, they do not appear to have been of much benefit; and I can well recollect the horrible pain and irritation which I suffered on one occasion from their application. I would trust principally to an assiduous use of the Goulard lotion and laudanum along the arm.

If the patient be in bad health at the time he receives the wound, there is danger of the local irritation increasing, so as to cause foul abscesses, similar to those formed in cases where a person with a bad constitution has been pricked by a nail, fish-bone, or oyster-shell. If these symptoms should take place, we must treat our patient in the same manner as we do that affection which is familiarly known by the term of the bad hospital-hand; and the best treatment for such cases is, I believe, nearly the same in principle as that already laid down to subdue the first attack. There will necessarily be considerable attention required to manage the abscesses, so as to prevent the tendons from sloughing. I have seen several cases where sloughing abscesses ensued, but happily they have all terminated well: there has not been even the motion of a joint lost.

I doubt whether any thing that is applied immediately on the injury being received, will prevent its bad effects, I have, during the dissection of bodies in every possible variety of condition, wounded my fingers many hundred times, and I have only suffered severely twice. I sometimes took measures to guard against the effects, but I have more frequently entirely neglected the wound: thus we lose all proof of what will produce or prevent the bad consequences; but still I would recommend that the student be careful while he is dissecting putrid ligaments, or preparing bones which have been macerated. If he cuts himself while thus engaged, he should encourage the part to bleed, and, after washing, suck it for some time. I have not much confidence in caustic as a preventive, and I have seen great irritation arise from its use.

I may just allude to the cases given by Dr. Duncan, of Mr. Whitelaw, of Mr. W. D., and of Mr. A. B. The history of the symptoms in each of those cases corresponds with what I have observed when a wound has been received in the dissecting room; but it is my duty to state my belief, that the symptoms would have been more quickly subdued, had a treatment similar to that I have recommended been pursued.

The other class of cases is more serious, as it constitutes an affection to which the practitioner is more liable than the student, and one that is attended with immediate danger to life.

All who have been in the habit of attending to morbid anatomy, must admit that there is more danger from a wound received during the examination of the body of a person who has died from any form of inflammation of the peritoneum, than of any other disease. So much have I been convinced of the truth of this, that I have for many years inculcated on the student the necessity of being particularly careful when examining a body, even though it is not putrid, where death has been caused by puerperal fever, the operation for hernia, or any form of peritonitis, or even of pleuritis.

If, five or six hours after examining such a body, pain is felt in the finger, and a small pimple or a blush of red is found at the part, immediate attention should be paid to it. If the case proceeds in the usual manner, there will probably be a darting pain up the arm, which seems to fix more particularly in the shoulder or side of the chest. Within fourteen hours, the patient is very ill; he suffers a great deal of pain, and is anxious and alarmed. Red lines may generally be perceived running from the hand towards the axilla, but it sometimes happens that there are no marks on the arm, nor even on the finger. Indeed, the affection of the finger is occasionally so slight, that it is neglected, and the patient refers all his suffering to the shoulder and chest. I have known two instances where the affection was mistaken for acute rheumatism. I shall quote from Dr. Duncan's paper, the history of the cases of the lamented Dr. Dewar, of Edinburgh, and of Mr. Hercey, in illustration of the rapid and insidious nature of those attacks.

"Dr. Dewar, a resident Fellow of the Royal College of Physicians of Edinburgh, wounded a finger of the left hand slightly, when examining the body of an enteritic patient, on Monday, the 13th of February, 1823, and immediately applied caustic to the wound. He attended a meeting of the Royal Society in the evening. On Tuesday he remained in bed, complaining of languor, general debility, and affected with rigors. On the 16th, the left axilla became exceedingly painful, without much swelling of the part, or any hardness in the course of the absorbents on the arm. Leeches were applied to the axilla, with great relief to the pain. On Saturday, at eight A.M., one of his medical friends was sent for, on account of an accession of pain in the right fore-arm, with some swelling and redness. Leeches were immediately applied, which bled freely, but without affording any relief; for the inflammation and swelling extended rapidly. At five P.M. he was bled from a vein in the arm, and the blood was sizy, without much separation of serum. Early in the morning of the 19th, he became insensible, and he died at nine A.M. The symptoms throughout were those commonly called typhoid, and the treatment strictly antiphlogistic.

"Body not examined."

"John Hercey, esq. aged thirty-four, residing in the Royal Infirmary, and senior physician's clerk, received, on Sunday, 25th August, 1821, a puncture in the ring-finger of the left hand, when stitching up a dropsical subject, which he had been assisting to dissect, within twenty-four hours of the death of the patient.

"During the afternoon of the following day he complained a little, and at night was seized with severe rigors, accompanied by thirst, prostration of strength, and other symptoms of constitutional irritation. Twenty leeches were applied to the arm, from the elbow upwards, and afterwards warm fomentations and poultices.

"27th, noon.—Complains of considerable head-ache and sickness, with perceptible though slight oppression, and anxiety of breathing; countenance somewhat collapsed, and expressive of anxiety. Pulse frequent; bowels opened by medicine. Does not complain much of pain in the finger, but a pustule has appeared where the puncture was made, and a distinct blush of redness, of about an inch or an inch and a half in breadth, extending from a little below the elbow to near the arm-pit. A circumscribed swelling has also taken place, embracing the arm around the elbow, and extending a little above and below the joint, but without any defined margin.

"Notwithstanding he suffered much from his symptoms and constitutional irritation, he attended Dr. Hamilton, and officiated at the visit.

"28th, 10 A.M.—Passed a restless night. Swelling considerably increased, and the redness is spread over nearly all the extent of the tumified part, being evidently of an erysipelatous blush. The finger is nearly in the same state as yesterday. The affection of the breathing rather increased. Pulse frequent; thirst urgent. Countenance more anxious; but is out of bed, and insists upon officiating at the visit at noon, although evidently very unfit for any exertion.

" 29th.—Arm continues to increase in size, and inflammation to spread in both directions from the elbow. The sore on the finger is of little consideration, giving no pain or uneasiness; no distinct trace of inflamed lymphatics can be perceived extending along the fore-arm, and there is no tumefaction, or pain of the glands in the axilla. Has had retching and vomiting of bilious matter repeatedly since last night. Respiration anxious, and somewhat catching, but denies having any pain in the region of the chest. Pulse rapid; thirst continues urgent. Anxious expression of countenance more marked, with evident sinking of features. Cloths, wet with solution of sugar of lead and tincture of camphor, have been kept at the arm constantly for the last two days.

" 30th.—Restless night. Has had nausea; symptoms continue; swelling extending, and redness spreading.

" 31st.—Symptoms unabated. Is recommended to leave the hospital, and to go to private lodgings; to which he was removed accordingly.

" Sept. 1st.—Inflammation and swelling of the fore-arm increased, extending to the shoulder, and down towards the wrist. Several vesicles, some of an oblong, others of a more circular shape, appeared to-day on the back of the fore-arm, below the elbow, and along the outer and inner side of the arm, above the joint; the smallest of which is about the size of a sixpence, elevated, and the longest from two to three inches in length. Some are filled with transparent serum, and others with a thin fluid, but of a dark colour. The erysipelatous inflammation encircling these vesicles has assumed a dusky hue, which extends over the greater part of the arm, from above the wrist, and upwards over the shoulder; but becomes gradually fainter as it recedes from the bases of these vesicles. Thirst urgent; pulse frequent, and febrile symptoms aggravated.

" 2d.—Very restless night, but declined taking an opiate, which had been prescribed. Considerable nausea to-day, especially after taking liquids. Bowels have been opened by medicine. Inflammatory hue, deepening in tint, extends over the deltoid muscle, and from the breast backwards over the shoulder. The region of the deltoid is also occupied by vesicles similar to those already described, varying in size, and filled with a dark serous fluid. Some of the vesicles on the arm were opened, and some have burst. The swelling has diminished in the lower part of the fore-arm; but, on the whole, the symptoms are in no respect improved.

3d.—State of the arm not improved. Most of the vesicles have burst, and have discharged such quantities of fluid as to penetrate the bed, and run upon the floor. Had an obvious tendency to delirium during the night, and to-day is distinctly observed to waver occasionally. No improvement in any respect.

" 4th.—Had a very restless night, with frequent copious vomiting of a dark-coloured matter; but about five in the morning he called for his breakfast, conversed with his landlady, said that he never should forget her attention, and ate to all appearance with an appetite; but expired at half-past six A.M.

"*Treatment.*—The solution was continued to the arm; a few laxative pills were given. On the evening of 1st September, a little claret was ordered, of which he accepted with reluctance, but the nurse persevered in giving him a little occasionally. On the evening of the 2d September, a grain of solid opium was prescribed. Had a little tea and panada for food, and barley-water for drink.

"Body not examined."

In our difficulty of explaining the cause of such symptoms as those unfortunate gentlemen suffered from, and which bear a close resemblance to other cases that have occurred within these few years, we cannot avoid considering them as in some respects resembling the effects of a specific poison; and the analogy is made still stronger by the sudden and tense tumefaction which rapidly extends from the elbow to the neck,—a swelling which, to the inexperienced eye, gives the idea of matter being formed deep under the skin, and has much resemblance to that which John Bell, in his description of the hospital gangrene, has called "soft and boggy, as if there were matter under it." I remember having been much struck with the resemblance in the appearance of the leg and thigh of a boy who had been bit by a viper, to that of a limb in this condition. I have been told that vesications often appear on the limb, from the bite of a venomous serpent. The same appear after the wounds received in dissection, and, in those cases I have seen, they were so nearly similar to those which are seen on a putrid body, that we cannot be surprised at the alarm of the student. But sometimes all the swelling subsides, and there is merely desquammation of the cuticle. When suppuration ensues, the matter is foul and fetid, and there is often extensive sloughing.

The wounds received in the dissecting-room resemble those vulgarly called poisoned wounds, (which occur from a variety of causes in hospitals;) but, after being wounded in the examination of a body recently dead, the whole system seems to be under the influence of a specific poison, which acts in a remarkably severe and rapid manner. This is not, however, the opinion commonly received: it is not supposed that the bad symptoms arise from the absorption of a specific poison, but merely from irritation. Now, while we admit that the prick of a clean needle or of a new lancet will, in some constitutions, cause symptoms nearly similar to those described above, we cannot deny the fact that several individuals (and, as we may presume, in different states of health,) may be affected by touching the same body.

So far I have found it necessary to enter into the theory of this affection, for upon these views I have acted in practice. It has happily not fallen to my lot to see many cases of this kind; and, of the few I have seen, none have proved fatal.

With regard to the treatment, the most important question seems to be what is to be done during the first twenty-four or forty-eight hours? for, after this time, the peculiar character of the disease is in a great measure lost. The patient may fall into a low typhoid state, or he may labour under the symptoms of the erysipelas phlegmonoides; so that, after a few days, there may be a question as to whether he is to be stimulated and supported, or to be reduced by bleeding.

Are we to bleed in the first instance?—Is a man who is suffering under a poison to be bled, because he has a pulse at 140, rather hard and full, head-ache, anxiety and difficulty of breathing, and flushing of the face? Under almost any other circumstances, I believe no one would for a moment doubt the propriety of a copious bleeding, when such symptoms are present: but may not the pulse be raised by a morbid irritation,—the anxiety and flushing of the face proceed from terror,—the head-ache from the same cause,—and the difficulty of respiration be owing partly to the same, and to the morbid condition of the principal respiratory muscles? But we seldom have the face flushed; it is generally pale and haggard, with a cold perspiration on the brow; and the tongue has not the white dry surface, nor has the pulse the hard stridulous beat, that marks the inordinate action which requires to be subdued by blood-letting. Proceeding on these views, and on the conclusions I have drawn from the accounts given of the effects of bleeding in the cases that are recorded, I have objected to venesection. I have also objected to local bleeding by leeches; for the swelling and inflammation of the arm is of a character that does not appear to be benefited by such treatment; and although, from the cases recorded, we may conclude that local bleeding has been sometimes of advantage, still its good effects have not been sufficiently marked to induce me to run the risk of reducing in this way the vital energies of my patient.

I shall here give the outline of a case that occurred last spring. About two in the afternoon, one of our pupils examined the body of a person who died of puerperal fever. He dined in the City; but was obliged to come home before eight o'clock, as he was suffering excessively from pain in his finger. He was not aware that he had pricked it during the dissection, but he found a little abrasion of the skin by the side of the nail. The pain increased so much, that he could not sleep, and he walked about the room in great agony during the greater part of the night. I saw him in the morning: he had all the general symptoms that are so well described in the cases I have quoted from Dr.

Duncan's paper. I immediately gave him a smart dose of calomel and colocynth, with a little antimony, and ordered the arm and hand to be kept constantly covered with pledgets, dipped in the opiate and lead lotion. I dosed him also well with laudanum. By this treatment the pain in the finger subsided, but the swelling of the arm and shoulder still increased, and towards the evening he suffered much from pain in his side. He was naturally very abstemious, and, as he had the day preceding taken a few glasses of wine at dinner, which he was not accustomed to, I could not prevail on him to take as much porter as I wished. I pursued the same treatment during the next day, combining the calomel and opium, with the intention of affecting his mouth. As he had notions of his own on the nature of such cases, I could not induce him to take all the cordials I proposed, in the form of negus, &c. On my visit in the afternoon, at his earnest request, I cut fairly down to the bone at the part where the finger was discoloured, but I found no matter. The pain had, previously to my making this cut, subsided in a great degree. After this, the wound of the finger became more of the character of a common sore. I saw him in the evening, and ordered him to go on in the same manner; but I was called to him again at midnight: he was then suffering greatly from head-ache, from difficulty and anxiety in respiration, with pain over the chest. His pulse was full, and at 140 in the minute. I was now in much difficulty, for I saw that he was in great danger, and his symptoms such that, if I called a consultation, my objections to his being bled would be over-ruled; but, as I had a strong conviction that bleeding would not relieve him, I determined to take the responsibility on myself; and I was strengthened in the view I took of his case, when I saw that his face was haggard, that he was rather chilled than heated, that he was in great alarm, and that his mental agitation had been increased by his making his will during the evening. Under these circumstances, and notwithstanding the state of his pulse and the head-ache, I gave him thirty-five drops of laudanum,—tried to persuade him that his alarm was needless,—and left the same quantity of laudanum to be taken in four hours afterwards, if the symptoms continued. He had no occasion to take it: the next morning, the head-ache and difficulty of breathing had disappeared, and from this time he gradually recovered; the calomel and opium being continued until his mouth was a little affected. During this time he took wine and porter pretty freely. He was naturally of a bad constitution, but no abscesses formed in the arm; notwithstanding which, he suffered much from an erysipelatous erythema, which extended over all the parts which had been swollen.

The symptoms in this case at first were exactly similar to several of those which have terminated fatally; the patient was also of a very bad constitution: I trust, therefore, that the short sketch I have given of the mode of practice pursued will be considered worthy of notice. The question of the propriety of bleeding in the first instance appears to me to be the most important. I believe I am at issue with several of my friends on this point. There may certainly be symptoms absolutely requiring the loss of blood; but I think there is much analogy between the abortive attempts to reduce the *boggy* swelling of the arm by running a lancet into it, and the hope of relieving the anxiety and the difficulty of respiration by general bleeding.

By way of defending the fingers when examining the abdomen or thorax, I generally anoint my hands with lard or pomatum. After the numerous fatal instances that have occurred of late years, no one should be accused of effeminacy, even if he were to wear thin gloves during the examination of a body of a person who had died of peritonitis. It is difficult to say what is the best thing to do when the finger is wounded under these circumstances. Washing it well with soap and water, afterwards sucking it, and then bathing it in spirits or turpentine, would probably prevent any bad results. But, if this should be ineffectual, the moment the pain commenced, I would immerse my finger in equal parts of laudanum and Goulard's lotion; I would take a smart dose of calomel and antimony, and two hours afterwards a large dose of laudanum. If the pain still continued, I would bathe my whole hand and fore-arm with tepid laudanum and Goulard, take another opiate, perhaps with a little ammonia, and even force myself to swallow cordials in the form of warm negus, &c.*

*Berner-street; April 12th,* 1825.

---

* On conversing lately with some of my professional friends on the subject of this paper, I found that objections were made to some of my views, but particularly to the recommendation of generous diet and porter. I am ready to admit, with regard to this last, that I have, to a certain extent, acted empirically, having been guided by the relief experienced by myself and others, when labouring under great pain. Although I do not fully understand the nature of the fever consequent on wounds received in dissecting, I maintain, nevertheless, that the symptoms (whether dependent on the absorption of a specific poison or not,) are such as indicate stimulants and narcotics, rather than depletion. It is with a view to its narcotic effects, that I recommend the porter: at the same time I would by no means persevere in its administration, if the symptoms appeared aggravated by it; but this, I repeat, I have not found to be the case.—AUTHOR.

*** We beg leave to refer to a paper on this subject by Dr. A. T. THOMSON, which will be found in another part of this Number.—EDITORS.

